# An Appearance-Semantic Descriptor with Coarse-to-Fine Matching for Robust VPR

**DOI:** 10.3390/s24072203

**Published:** 2024-03-29

**Authors:** Jie Chen, Wenbo Li, Pengshuai Hou, Zipeng Yang, Haoyu Zhao

**Affiliations:** School of Mechanical Engineering and Automation, Northeastern University, Shenyang 110819, China; chenjie@me.neu.edu.cn (J.C.); 2170170@stu.neu.edu.cn (W.L.); 2270262@stu.neu.edu.cn (Z.Y.); 2300500@stu.neu.edu.cn (H.Z.)

**Keywords:** visual place recognition, appearance-semantic information fusion, coarse-to-fine matching strategy, semantic segmentation

## Abstract

In recent years, semantic segmentation has made significant progress in visual place recognition (VPR) by using semantic information that is relatively invariant to appearance and viewpoint, demonstrating great potential. However, in some extreme scenarios, there may be semantic occlusion and semantic sparsity, which can lead to confusion when relying solely on semantic information for localization. Therefore, this paper proposes a novel VPR framework that employs a coarse-to-fine image matching strategy, combining semantic and appearance information to improve algorithm performance. First, we construct SemLook global descriptors using semantic contours, which can preliminarily screen images to enhance the accuracy and real-time performance of the algorithm. Based on this, we introduce SemLook local descriptors for fine screening, combining robust appearance information extracted by deep learning with semantic information. These local descriptors can address issues such as semantic overlap and sparsity in urban environments, further improving the accuracy of the algorithm. Through this refined screening process, we can effectively handle the challenges of complex image matching in urban environments and obtain more accurate results. The performance of SemLook descriptors is evaluated on three public datasets (Extended-CMU Season, Robot-Car Seasons v2, and SYNTHIA) and compared with six state-of-the-art VPR algorithms (HOG, CoHOG, AlexNet_VPR, Region VLAD, Patch-NetVLAD, Forest). In the experimental comparison, considering both real-time performance and evaluation metrics, the SemLook descriptors are found to outperform the other six algorithms. Evaluation metrics include the area under the curve (AUC) based on the precision–recall curve, Recall@100%Precision, and Precision@100%Recall. On the Extended-CMU Season dataset, SemLook descriptors achieve a 100% AUC value, and on the SYNTHIA dataset, they achieve a 99% AUC value, demonstrating outstanding performance. The experimental results indicate that introducing global descriptors for initial screening and utilizing local descriptors combining both semantic and appearance information for precise matching can effectively address the issue of location recognition in scenarios with semantic ambiguity or sparsity. This algorithm enhances descriptor performance, making it more accurate and robust in scenes with variations in appearance and viewpoint.

## 1. Introduction

In recent years, the advancement in computer vision has brought significant attention to the task of visual place recognition (VPR). VPR aims to identify and match locations using image information accurately, and it is of great significance for applications such as visual localization and mapping, loop closure detection, driving, robot navigation, and augmented reality. However, VPR faces challenges due to variations in appearance and viewpoint.

Traditional handcrafted appearance-based features, such as SURF [1] and ORB [2], have been widely used in visual place recognition (VPR). However, these algorithms suffer from performance degradation when faced with changes in appearance and viewpoint. Some end-to-end VPR algorithms, including NetVLAD [3], have been developed to address this problem by extracting robust image features using deep neural networks. Nevertheless, the generalization ability of end-to-end networks remains questionable, and they require a substantial amount of training data.

In contrast, the semantic information of images is relatively unaltered. Therefore, numerous studies have utilized semantic information to build more robust VPR algorithms. For example, stable feature representations can be obtained by encoding images using semantic segmentation boundaries [4,5]. This algorithm is, nevertheless, susceptible to the quality of the object segmentation.

Other studies have utilized random walk algorithms to transform the semantic information of images into a 3D graph structure to obtain a more complete semantic representation [5,6]. Although these algorithms can represent relatively complete semantic information in images, they have high computational resource requirements and complexity. In practical applications, VPR algorithms need to not only deal with various potential changes, but also meet the requirements of real-time positioning and navigation systems. Therefore, it is necessary to design a lightweight image descriptor to achieve robust VPR tasks. The Forest [7] descriptor has made significant progress in terms of being lightweight, efficient, and robust. While the Forest descriptors are capable of capturing semantic information in a scene, they may encounter challenges such as semantic information overlap and sparsity in extreme scenarios. Thus, additional constraint information is needed to enhance the performance of the descriptor further.

To address this problem and further enhance the performance of the descriptor, this paper proposes a descriptor based on both appearance and semantic information, aiming to extract robust appearance information and tightly couple it with semantic information. Our algorithm has made significant progress in terms of being lightweight, efficient, and robust. Specifically, we combine SuperPoint feature points [8] and semantic images to capture appearance information and use semantic contours to classify and cluster feature points [9], generating a VLAD vector representing each contour. Ultimately, appearance information is assigned to every semantic object by combining the Forest descriptor, yielding a new appearance semantic descriptor known as the SemLook local descriptor. To further improve the algorithm’s accuracy, we introduce a preliminary screening strategy based on semantic contours and use it to construct the SemLook global descriptor. In summary, this paper makes the following contributions:

- This paper proposes an algorithm for generating descriptors that integrate image appearance and semantic information, thereby tightly coupling appearance and semantic information to generate new appearance semantic descriptors. This fusion enhances the expressive power of the descriptors, leading to improved accuracy and robustness in position recognition.
- This paper proposes a coarse-to-fine image matching strategy. The semantic contour is first used to construct the SemLook global descriptor for initial screening. Then, appearance semantic descriptors, namely, SemLook local descriptors, are introduced to obtain more accurate position recognition results. This strategy further improves the accuracy and robustness of position recognition.
- Our proposed algorithm is compared with six state-of-the-art VPR algorithms on three public datasets with appearance and viewpoint changes. The results demonstrate that the SemLook-based VPR approach achieves competitive performance in terms of robustness, accuracy, and computation consumption.

## 2. Related Work

In this section, we review the current state-of-the-art techniques for visual place recognition (VPR). Traditional VPR algorithms mainly rely on appearance-based descriptors, such as visual words and interest point detectors. These algorithms, like FAB-MAP [10], usually use feature points for scene matching; however, their performance is limited in situations where there are significant variations in the scene. To address the issue of scene variations, some algorithms have adopted clustering techniques, such as DBoW [11] and IBoW-LCD [12], assigning feature points in the image to predefined cluster centers and then representing the image by computing the residual vector of each cluster center. Another appearance-based algorithm is CoHOG [13], which utilizes efficient image descriptors based on the histograms of oriented gradients (HOG [14]), and uses region-of-interest extraction and convolutional matching algorithms to maintain robust performance. However, these algorithms suffer from reduced performance when faced with external factors such as changes in appearance and viewpoint.

With the development of neural network technology, convolutional neural networks (CNNs) have been applied to visual place recognition (VPR) tasks by extracting features and optimizing performance. The application of CNNs in VPR was first pioneered by Chen et al. [15], and subsequent studies have continuously utilized CNNs to extract image features. One important direction is addressing environments with significant lighting variations. Hou et al. [16] used the AlexNet [17] model, a classic CNN architecture known for its robustness to lighting changes, which helps to extract more reliable features. Another breakthrough algorithm is NetVLAD, which introduces a new VLAD layer, enabling an end-to-end VPR approach. NetVLAD aggregates feature vectors extracted by CNN into global descriptors and performs remarkably in VPR tasks. To optimize local features, researchers have developed the PatchNetVLAD [18] algorithm, which focuses on extracting local features and improving the NetVLAD model. PatchNetVLAD significantly enhances robustness and recognition accuracy by combining local and global features. AMOSNet [19] transforms visual place recognition into a classification problem using neural networks. Inspired by transfer learning, HybridNet [20] re-trains the weights of the convolutional layers’ initialization, resulting in an improved version of AMOSNet. Additionally, RegionVLAD [21] is an efficient algorithm that extracts region features from intermediate layers of CNN. It utilizes feature maps extracted by CNN at multiple scales to compute descriptors for local regions and generates global descriptors through pooling operations, ensuring invariance across various environments.

However, using CNNs requires a large amount of model training and labeled datasets, and their generalization ability is relatively poor, lacking robustness to extreme viewpoint changes. Therefore, some researchers have attempted to introduce semantic information to improve VPR algorithms. Some algorithms combine semantic segmentation results with feature maps extracted by CNN to generate global descriptors. For example, LOST-X [22] integrates feature maps extracted by CNN with semantic segmentation results, achieving excellent VPR performance. Other algorithms utilize semantic graph models to represent scenes with semantic segmentation results and incorporate odometry information to build 3D maps, such as X-view [6] and algorithms proposed by Guo [23] et al. These algorithms excel in improving robustness and stability. However, the computational complexity of these algorithms is relatively high, limiting their widespread application in practical scenarios. This led to the development of Forest, and Forest descriptors have made significant progress in terms of being lightweight, efficient, and robust. Forest descriptors can capture the geometric relationships between semantic objects in a scene; however, they may encounter issues such as semantic information overlap and sparsity in extreme scenarios. Thus, additional constraint information is needed to enhance the performance of the descriptors further.

In summary, traditional appearance-based VPR algorithms have certain limitations. In contrast, improved algorithms that incorporate deep learning and semantic information have, to some extent, enhanced the performance and robustness of visual localization. However, challenges and room for improvement still exist. Therefore, in this paper, we propose a novel appearance semantic local descriptor called SemLook, building upon the foundation of Forest semantic descriptors. By effectively integrating the semantic and appearance information of local images, SemLook achieves improved accuracy and robustness. Additionally, to further enhance the accuracy of the descriptor, we introduce a new strategy called the semantic contour-based pre-filtering strategy. The SemLook global descriptor constructed based on this strategy utilizes semantic contours to perform initial filtering of images, which helps to improve the accuracy and reliability of visual localization tasks.

## 3. Algorithms

To enhance the robustness and accuracy of the algorithm, we adopted a coarse-to-fine strategy for image matching. The core idea is to utilize semantic contours to construct SemLook global descriptors, which are used for the initial filtering of images. Then, based on the local descriptor, the candidate frame set obtained by filtering is further filtered, and finally, the correct matching result is obtained. The global descriptor of SemLook contains the structural distribution of semantic information, which can effectively eliminate false recognition, helping us to quickly narrow the matching range and improve the matching efficiency. The SemLook local descriptor assigns appearance information to each semantic object in the Forest descriptor, thus better understanding the appearance characteristics of semantic objects while grasping their geometric relationships and ultimately providing more accurate matching results.

Overall, the integration of SemLook’s global and local descriptors has produced more precise matching outcomes, leading to increased image matching robustness and accuracy. The application of this approach is of significant importance for improving the performance of image matching algorithms and has achieved satisfactory results in practical applications.

### 3.1. SemLook Global Descriptor

**Figure 1:**
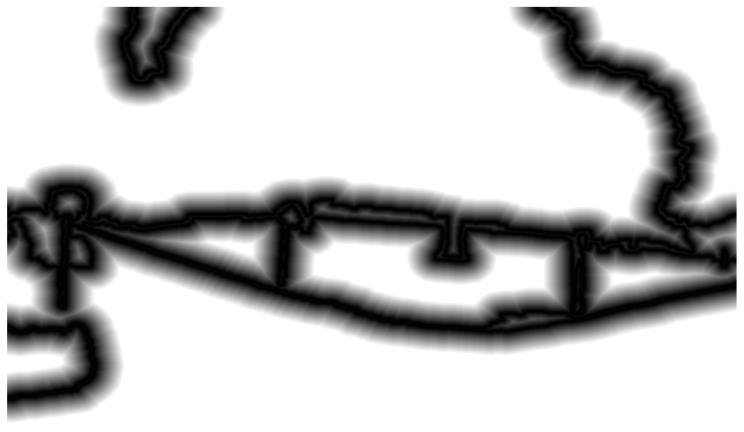
Semantic contour distance field diagram.

We used a distance field generation algorithm based on semantic contours when constructing image descriptors. Specifically, we employed the free region growing algorithm to generate the distance field, as shown in Figure 1. This algorithm calculates the distance information between each pixel and the nearest semantic contour by continuously growing the boundary of the contour. The distance field records the distance value between each pixel and the nearest semantic contour [24].

The process of generating the distance field is as follows: First, we select the boundary points of the semantic contour as initial points and mark them as contour points. Subsequently, we calculate the distance between the neighboring pixels and the contour point and select the closest pixel as the current pixel. Next, we mark the selected pixel as a contour point and calculate the distance between its neighboring pixels and the contour point. We repeat these steps until all pixels have been visited and marked as contour points. We record the distance value between each pixel and the nearest contour point during this process. Using this free region growing algorithm, we obtain the distance information between each pixel and the nearest contour, forming a distance field. The specific calculation Formula (1) is as follows:

(1) $$D\left(P\right)=min\left\{d\left(P,\;C\right)\right\}$$

In the equation, P represents a pixel in the image and C is a set of points on the semantic contour. $d\left(P,\;C\right)$ denotes the Euclidean distance between the point P and any point in the set C.

To enhance the stability of the distance field, this study adopted a post-processing method to reduce noise in edge extraction and edge changes caused by viewpoint variations. Specifically, we set a threshold, typically one-twentieth of the minimum of the image’s width and height. In the post-processing phase, if the distance value of a pixel exceeded this predefined threshold, the distance value of that pixel was reset to zero. This step aimed to eliminate potential noise or unstable distance values and mitigate the effects of viewpoint changes, thereby improving the reliability and accuracy of the distance field. Through this processing, we obtained a global descriptor that reflected the semantic structural features of the image.

### 3.2. Local Descriptors in SemLook

#### 3.2.1. Original Forest Architecture

The original Forest descriptor is a lightweight semantic-based image descriptor that utilizes only two-dimensional image information for high-precision visual place recognition (VPR) and exhibits a certain robustness to variations in viewpoint and appearance. The specific algorithm is as shown in Algorithm 1.
**Algorithm 1** Semantic Object Contour Extraction and Forest Descriptor Encoding**Input:** semanticSegmentationResult **Output:** Forest descriptorInitialize contours as an empty list**for** each unique semantic category in semanticSegmentationResult **do**   Extract pixels belonging to the current semantic category   Layer the image based on the semantic category   Binarize each layer   Extract contours from each binarized layer   Add extracted contours to contours**end for**Initialize trees as an empty list**for** each contour in contours **do**   Extract semantic category of the contour    Calculate center position of the contour    Calculate area of the contour   Initialize neighborScores as an empty list   **for** each other contour in contours **do**      Calculate geometric relationship with the current contour      Add the score and index of the neighboring contour to neighborScores   **end for**   Sort neighborScores based on the scores   Select indices of the top three scores from neighborScores as neigh-borIndices   Initialize localDescriptors as an empty list   **for** each index in neighborIndices **do**      Add semantic category of the contour at index to localDescriptors   **end for**   Create a tree with semantic category, center position, area, and localDe-scriptors   Add the tree to trees**end for**Set Forest to the collection of all treesReturn Forest

It is worth noting that in the semantic segmentation result, pixels belonging to the same semantic category form multiple closed regions. Semantic objects are extracted based on these closed regions. Then, the image is layered according to semantic categories, and each layer is binarized. Finally, the contour of each binarized layer is extracted, obtaining the contours of all semantic segmentation objects in the image [25].

**Figure 2:**
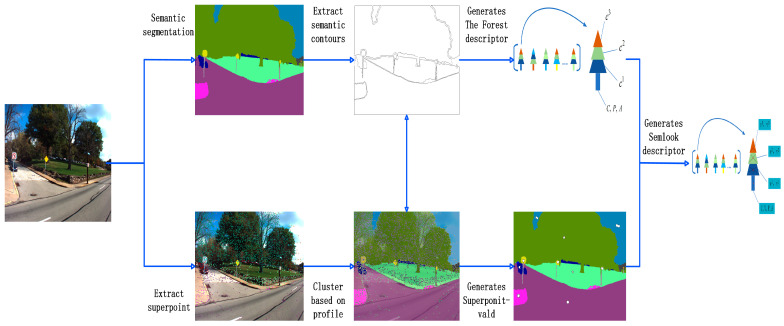
SemLook local descriptor-encoding process.

We introduced appearance information to enhance the expression power of the Forest descriptor. Based on the Forest descriptor, we extracted SuperPoint feature points from the image according to appearance information and extract contours for each semantic object based on the semantic segmentation of the image. SuperPoint feature points were classified according to the semantic contours, and the SuperPoint-VLAD representing the appearance information of each contour was generated by clustering the SuperPoint feature points inside each semantic contour. The appearance information obtained was assigned to each semantic object in the Forest to obtain the new appearance semantic descriptor SemLook. Figure 2 shows the process of encoding the SemLook descriptor for the image.

#### 3.2.2. SuperPoint-VLAD

**Figure 3:**
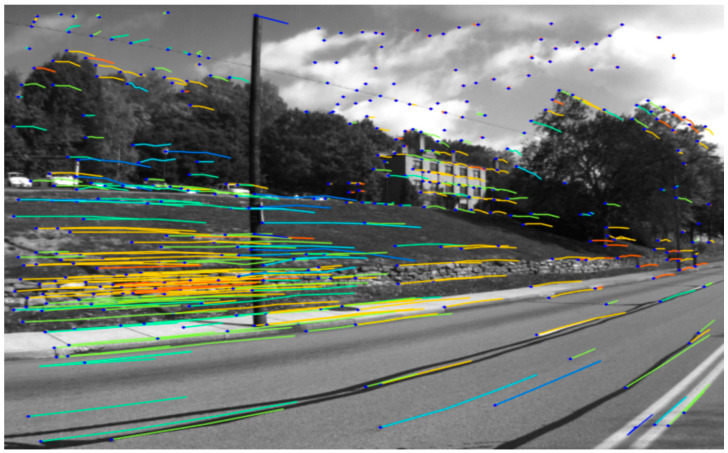
SuperPoint: image interest points detection and description.

SuperPoint is a self-supervised learning method for detecting and describing image interest points. The main idea behind SuperPoint is to train a neural network to learn the ability to extract appearance information from images automatically. It can identify salient corner points, edges, and key points, as shown in Figure 3, which exhibit good appearance consistency and repeatability. These points can be applied to various computer vision tasks such as image matching, tracking, and pose estimation. By leveraging the learned representations of appearance, SuperPoint provides a robust and efficient approach for dealing with interest point detection and description tasks in images.

SuperPoint is robust in terms of detecting and describing points of interest in complex environments under the conditions of noise, occlusion, illumination change, and viewpoint change. Therefore, it can improve the understanding of appearance information for visual localization to some extent, enhancing its performance and robustness.

VLAD (vector of locally aggregated descriptors) is an algorithm for image feature representation that integrates local feature descriptors into a global vector representation based on clustering and coding techniques. The VLAD algorithm can effectively capture spatial layout and appearance information in images.

SuperPoint-VLAD combines the SuperPoint feature detector and the VLAD algorithm to extract appearance information for each semantic contour in an image. By leveraging the SuperPoint algorithm to extract robust feature points and the VLAD algorithm to encode these feature points, SuperPoint-VLAD can generate global vector representations that capture appearance information for each contour. The formation process of Super-Point-VLAD is illustrated in Algorithm 2.
**Algorithm 2** Compute SuperPoint-VLAD for Contours**Input:** contours, keypoints1, descriptors1 **Output:** representations for each contourInitialize SuperPoint-VLAD as an empty list**for** each contour in contours **do**    Initialize centerx and centery to 0    Initialize ptnum to the number of points in contour    **for** each point P in contour **do**      Add P.x to centerx and P.y to centery    **end for**    Compute the center point ct as (centerx/ptnum, centery/ptnum)    Initialize keypointsInContour and descriptorsInContour as empty lists    **for** each keypoint kp in keypoints1 **do**      if kp is inside contour then        Add kp to keypointsInContour        Add the descriptor corresponding to kp to descriptorsInContour      end if    **end for**    if keypointsInContour is not empty then      Initialize min distance to infinity, min_index to −1      **for** each point kp in keypointsInContour **do**        Calculate the distance dist between kp and ct        if dist is less than min distance then          Update min distance to dist, min index to the index of kp        end if      **end for**      Set codebookDescriptor to descriptorsInContour[min_index]      Initialize vlad as a zero vector      **for** each descriptor desc in descriptorsInContour **do**        Calculate the difference diff between desc and        codebookDescriptor        Accumulate diff to vlad      **end for**      Normalize vlad with L2 norm      Add the normalized vlad to SuperPoint-VLAD    end if**end for**return SuperPoint-VLAD

Through the above process, the appearance information of each semantic contour is encoded as a global vector representation. This vector representation captures the distribution of key points and feature descriptors within the contour, providing comprehensive contour appearance information. The SuperPoint-VLAD algorithm effectively integrates SuperPoint features and the VLAD encoding process, extracting stable appearance features of contours, and is applicable to various image processing and computer vision tasks.

#### 3.2.3. SemLook Local Descriptor Construction

1. Extract the following information from the contours:

Semantic Category: The semantic category of the pixels contained in the contour represents the object’s category.

Center Position: The coordinates of the contour’s center represent the object’s position.

Area: Calculate the number of pixels in the contour as the object’s area.

Appearance: Calculate the SuperPoint-VLAD vector for each semantic contour as the object’s appearance.

2. For each object, we select the three most distinctive neighboring objects from its surrounding environment based on geometric relationships. Specifically, these neighboring objects are usually the ones closest to the target object in terms of geometric distance. In cases where multiple objects have similar distances, we prioritize those with larger areas. Subsequently, we integrate the semantic categories and appearance features of these neighboring objects into the local descriptor of the target object to capture the spatial relationships and visual features between objects. In this way, we can enhance the discriminative ability of the descriptor and improve its robustness in the face of scene changes.

In order to comprehensively calculate this geometric relationship, we use the following Formula (2) to consider the centroid distance and contour area between objects:

(2) $$\begin{array}{c} L_{ij}=\sqrt{{\left(P_{i}^{x}-P_{j}^{x}\right)}^{2}+{\left(P_{i}^{y}-P_{j}^{y}\right)}^{2}}\\ \delta_{ij}=\sqrt{L_{ij}^{\alpha }/A_{j}^{\beta }} \end{array}$$

Here, $P_{i}^{x}$, $P_{j}^{x}$, $P_{i}^{y}$, and $P_{j}^{y}$ represent the x. and y coordinates of objects i and j, respectively. $L_{ij}^{\alpha }$ represents the distance between the two objects, and $A_{j}^{\beta }$ represents the area of object j. α and β represent the weights of area and distance, respectively. $\delta_{ij}$ represents the geometric relationship between the two objects, where a smaller value of $\delta_{ij}$ indicates a stronger relationship.

We sort the three neighboring objects based on their scores and choose the semantic categories and appearance information of the three neighboring objects with the lowest A values as descriptors for the *i*th object, as shown in Formula (3). They are represented as $c_{i}^{1}$, $c_{i}^{2}$, and $c_{i}^{3}$ for the semantic categories and $v_{i}^{1}$, $v_{i}^{2}$, and $v_{i}^{3}$ for appearance, with superscripts indicating the order.

**Figure 4:**
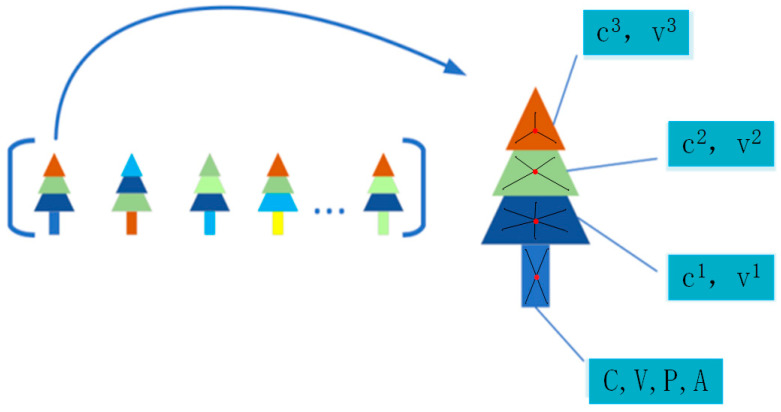
The Semlook descriptor (colors indicate semantic category).

3. We use the extracted information to encode each semantic object, obtaining a descriptor for each object, which is called a “tree”.
4. All the “trees” are then assembled into a collection, serving as the descriptor for the entire image, which is called the “Semlook” local descriptor and represented as *G*, as shown in Figure 4.

(3) $$G=\left\{T_{1},T_{2}\ldots T_{n}\right\}=\left[\begin{array}{cccc} C_{1} & C_{2} & \cdots & C_{n}\\ \begin{array}{l} V_{1}\\ P_{1} \end{array} & \begin{array}{l} V_{2}\\ P_{2} \end{array} & \begin{array}{l} \cdots \\ \cdots \end{array} & \begin{array}{l} V_{n}\\ P_{n} \end{array}\\ A_{1} & A_{2} & \cdots & A_{n}\\ c_{1}^{1}{,\;v}_{1}^{1} & c_{2}^{1}{,\;v}_{2}^{1} & \cdots & c_{n}^{1}{,\;v}_{n}^{1}\\ c_{1}^{2}{,\;v}_{1}^{2} & c_{2}^{2}{,\;v}_{2}^{2} & \cdots & c_{n}^{2}{,\;v}_{n}^{2}\\ c_{1}^{3}{,\;v}_{1}^{3} & c_{2}^{3}{,\;v}_{2}^{3} & \cdots & c_{n}^{3}{,\;v}_{n}^{3} \end{array}\right]$$

Here, $C_{n}$, $P_{n}$, $A_{n}$, and $V_{n}$, respectively, represent the semantics, center position, area, and appearance of the “tree”. $c_{n}^{1}$, $c_{n}^{2}$, and $c_{n}^{3}$ represent local descriptors of the tree, while $v_{n}^{1}$, $v_{n}^{2}$, and $v_{n}^{3}$ represent the appearance of the local descriptors. The formation process of Semlook local descriptors is illustrated in Algorithm 3.
**Algorithm 3** Construct Semlook Local Descriptor**Input**: contours, SuperPoint-VLAD **Output**: Semlook local descriptor GInitialize trees as an empty list**for** each contour in contours **do**    Extract semantic category of the contour    Calculate center position of the contour     Calculate area of the contour    Set appearance to the corresponding SuperPoint-VLAD vector    Initialize neighborInfo as an empty list    **for** each other contour in contours **do**      Calculate geometric relationship with the current contour      Add semantic category, appearance, and geometric relationship to neighborInfo    **end for**    Sort neighborInfo based on geometric relationship    Select top three objects from neighborInfo as localDescriptors    Create a tree with semantic category, center position, area, appearance, and     localDescriptors    Add the tree to trees**end for**Set G to the collection of all trees**Return** G

##### SemLook Global Descriptor Matching

SSIM is a measure of structural similarity between two images, which is widely used in the field of computer images. Inspired by the SSIM (structural similarity) measurement algorithm [26], this paper uses the following Formula (4) correlation coefficients to measure the similarity of global descriptors between query frames and reference frames in the matching stage.

(4) $$\rho (X,Y)=\frac{Cov(X,Y)}{\sigma (X)\sigma (Y)}=\frac{E(XY)-E(X)E(Y)}{\sigma (X)\sigma (Y)}$$

The calculation of covariance Cov(X,Y) is for the distance field X, generated by the semantic contour of the query frame, and the distance field X, generated by the semantic contour of the reference frame. E(XY) represents the mathematical expectation of the joint distribution of X and Y, while E(X) and E(Y) represent the mathematical expectations of X and Y, respectively.

Specifically, by calculating the correlation coefficient of the global descriptors between the query frame and the reference frame, the similarity score between them can be obtained. The correlation coefficient formula is a statistical algorithm used to measure the relationship between two variables. By calculating the correlation coefficient between two descriptors, we can evaluate their correlation and structural similarity.

Based on the computed similarity scores, we select the top N frames with the highest scores as the candidate frame set. The purpose of this is to choose the candidate frames that are most similar to the query frame during the matching process, enabling further analysis and processing.

##### SemLook Local Descriptor Matching

Obtain the SemLook local descriptors for the query image and reference image, represented as $G_{i}$ and $\tilde{G_{i}}$, respectively. For each “tree” $T_{i}$ in $G_{i}$, traverse all “trees” $\tilde{T_{i}}$ in $\tilde{G_{i}}$ and perform the following matching steps:

1. Compare the semantic categories C of $T_{i}$ and $\tilde{T_{i}}$. If the semantic categories are the same, calculate the similarity of their intrinsic information $s_{tree\;}$, which includes calculating the area similarity $s_{area\;}$, the center location similarity $s_{cent\;}$, and the appearance similarity $s_{look\;}$. The formulas for calculating the similarities are as follows:
  (5) $$s_{tree\;}=s_{area\;}s_{cent\;}s_{look\;}$$
  (6) $$s_{area\;}=\left\{\begin{array}{l} 1,if\frac{A_{i}}{\tilde{A_{i}}}>0.9\\ 0,if\frac{A_{i}}{{\tilde{A}}_{i}}<0.7\\ \frac{A_{i}}{{\tilde{A}}_{i}},\;others \end{array}\right.$$
  (7) $$s_{cent\;}=\left\{\begin{array}{c} \begin{array}{l} 1,if\;\tilde{L}<0.4\gamma \end{array}\\ 0,if\;\tilde{L}>0.7\gamma \\ 1-\frac{\left(\tilde{L}\;-\;0.4\;*\;\gamma \right)}{\left(0.3\;*\;\gamma \right)},\;others \end{array}\right.$$
  (8) γ=min(w,h)
  (9) $$s_{look\;}=\left\{\begin{array}{c} 1,\;if\;dist<5\\ \begin{array}{l} 0.75,if\;dist<14\\ 0.5,if\;dist<23 \end{array}\\ 0,\;others \end{array}\right.$$
  (10) $$dist=\sum_{j=1}^{128}|desc_{i}^{j}-{\tilde{desc}}_{i}^{j}|\;$$
  where $A_{i}$ and $\tilde{A_{i}}$ represent the area ratios of the two “trees”; $\tilde{L}$ represents the distance between them; γ is the minimum width and height of the input image size; $desc_{i}^{j}$ and ${\tilde{desc}}_{i}^{j}$ represent the SuperPoint-VLAD values of the image set and query set, respectively; and dist represents the Manhattan distance value of the SuperPoint-VLAD between the image set and query set.

Further, measure the similarity between $T_{i}$ and $\tilde{T_{i}}$ based on the similarity of their local descriptors. Compare the similarity of the local descriptors $c_{i}^{1}$, $c_{i}^{2}$, and $c_{i}^{3}$ of $T_{i}$ with the local descriptors $\tilde{c_{i}^{1}}$, $\tilde{c_{i}^{2}}$, and $\tilde{c_{i}^{3}}$ of $\tilde{T_{i}}$. If they have the same semantic category and similar appearance, they are considered a match, and the local appearance similarity $s_{locallook\;}$ is calculated using the following Formula (11):

(11) $$s_{locallook\;}=\left\{\begin{array}{l} 1,\;if\;dist<14\\ 0,others \end{array}\right\}$$

(12) $$dist=\sum_{j=1}^{100}|localdesc_{i}^{j}-{\tilde{localdesc}}_{i}^{j}|\;$$

where $localdesc_{i}^{j}$ and ${\tilde{localdesc}}_{i}^{j}$ represent the SuperPoint-VLAD values of the local descriptors of the image set and query set, respectively.

To guarantee that descriptors with the same order obtain better scores, adjustments must be made to their matching similarity scores due to the different geometric connections between, $c_{i}^{1}$, $c_{i}^{2}$, and $c_{i}^{3}$. We assign a weight of η to each element. Since there can be a maximum of three pairs of matches for local descriptors, each pair has a maximum score of 1/3. Hence, the matching score for the local descriptors of two “trees” can be calculated using the following Formula (13):

(13) $$s_{local\;}=\sum_{\kappa =1}^{n}\frac{\eta_{\kappa }\tilde{\eta_{\kappa }}}{3}$$

where $\eta_{\kappa }*\tilde{\eta_{\kappa }}$ represents the weight of the descriptor match pair, and n is the number of matched vertex pairs for local descriptors.

2. The similarity score S between two “trees” can be calculated using the following Formula (14):
  (14) $$s=s_{tree\;}s_{neighbor\;}$$
  where $s_{tree\;}$ is the self-similarity score of the object, and $s_{neighbor\;}$ is the similarity score of the object’s own local descriptors.

For each $G_{i}$, find the $G_{i}$ in $\tilde{G_{i}}$ that has the highest similarity score as the matching item. If the highest similarity score is zero, it means there is no match in $\tilde{G_{i}}$.

Calculate the similarity score of the entire image by computing the ratio of the number of matching “tree” pairs between $G_{i}$ and $\tilde{G_{i}}$ and the similarity score. The Formula (15) for calculating the overall similarity score is as follows:

(15) $$S=\frac{\sum_{v=1}^{m}s_{v}}{\max(M,N)}$$

where m represents the number of matching “tree” pairs, M and N represent the number of “trees” in the Forest descriptor of the image, and $s_{v}$ represents the similarity score for each pair of matching “trees.” The reason for taking their maximum values is that we believe that the number of “trees” contained in the image descriptor should be similar within the same scene. When there is a large difference in the number of “trees,” taking their maximum value can avoid incorrect matching.

##### Experiments

In this section, we evaluate the proposed algorithm on four different datasets and describe the details of the experimental setup.

##### Dataset

The first dataset used was Extended-CMU Season [27]. This dataset contains scenes with different lighting and seasonal conditions and records the pose information in the scenes. For this study, we selected sub-sequences named Slice6, Slice7, and Slice8 for experimentation. These sub-sequences represent urban road scenes with variations in seasonality and lighting and some changes in perspective, making them suitable for evaluating the robustness of visual localization and navigation algorithms to these variations. In constructing the ground-truth, we used a threshold of ±5 m, as stated in [28], to measure the difference between estimated poses and ground truth poses. Additionally, in terms of semantic categories, we analyzed eight semantic classes, including road, building, wall, fence, sky, vegetation, pole, traffic sign, and sidewalk.

**Figure 5:**
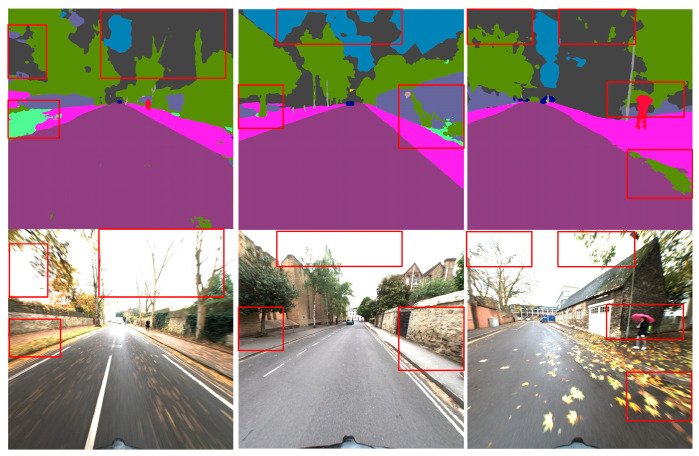
Examples of incorrect semantic segmentation in the dataset RobotCar Seasons v2.

The second dataset used was RobotCar Seasons v2 [29]. This dataset captures urban scenes in Oxford, UK, over a year, covering various changes in lighting, seasons, weather, and viewpoints. For this study, two sub-sequences were selected for experimentation: Sunny and Overcast–Winter. These scenes exhibit variations in lighting, seasonality, weather, and viewpoints, enabling an effective evaluation of the robustness of visual localization and navigation algorithms to these changes. Additionally, the images in this dataset possess characteristics such as motion blur and overexposure, resulting in significant semantic segmentation noise, as illustrated in Figure 5. Therefore, this dataset also serves to validate the robustness of our algorithm to semantic segmentation noise. We maintained a threshold of ±5 m to measure the error between estimated poses and ground truth poses while analyzing eight semantic classes, including road, building, wall, fence, sky, vegetation, pole, traffic sign, and sidewalk.

Another dataset used was the SYNTHIA-SEQS-02 [30] dataset. The SYNTHIA dataset is a synthetic dataset of urban scenes that includes pixel-level semantic annotated images. In this study, we used the foggy images from Sequence 2 of the SYNTHIA dataset as query images and the rainy night images and sunset images from the same sequence as reference images to validate the robustness of visual localization and navigation algorithms under varying lighting, seasonal, weather, and viewpoint conditions. Additionally, to evaluate the accuracy of visual localization and navigation algorithms, the dataset provides ground-truth data from VPR-bench [28]. We adopted ten classes defined in the SYNTHIA dataset for semantic categories, including buildings, roads, sidewalks, fences, vegetation, poles, lane markings, sky, traffic lights, and traffic signs.

We utilized a CNN model pre-trained on the cityscape dataset [31] to perform image semantic segmentation on the Extended-CMU Season and RobotCar Seasons v2 datasets [32]. For the SYNTHIA dataset, since pixel-level annotations are provided for each image, we directly used these pixel-level annotations to obtain the semantic segmentation results of the SYNTHIA dataset. Combining the pre-trained CNN model and existing pixel-level annotations, we successfully achieved the semantic segmentation tasks for Extended-CMU Season, RobotCar Seasons v2, and the SYNTHIA dataset.

##### Evaluation Indicators

In order to perform a fair evaluation of the proposed algorithm, we conducted three different experiments to systematically compare the accuracy, robustness, and computational cost of VPR techniques. We utilized the AUC (area under the C = curve) of the precision–recall curve, Recall@100%Precision, and Precision@100%Recall metrics to quantitatively assess the performance of each VPR algorithm in various scenarios.

1. AUC (area under the curve): The AUC metric measures the overall performance of a model by calculating the area enclosed by the precision–recall curve. A higher AUC value, closer to 1.0, indicates a more practical VPR algorithm with higher accuracy and robustness.
2. Recall@100%Precision: This metric represents the maximum recall achieved at 100% precision. It indicates the highest recall we can achieve while maintaining a precision of 100%. A value closer to 1.0 indicates better performance of the VPR algorithm under high precision.
3. Precision@100%Recall: This metric represents the precision achieved at 100% recall. In other words, it is the precision we can achieve while maintaining a recall of 100%. A value closer to 1.0 indicates better performance of the VPR algorithm.

By evaluating these metrics, we can objectively assess the performance of each VPR algorithm in different scenarios and determine their accuracy, robustness, and computational cost.

##### Experimental Setup

To evaluate the VPR performance of Semlook, we conducted three experiments on different datasets.

In the first experiment, we evaluated a number of cutting-edge VPR algorithms and compared them with our own approach. These algorithms included appearance-based algorithms such as HOG [14] and CoHOG [13]; CNN-based algorithms such as AlexNet_VPR [17], Patch-NetVLAD [18], and RegionVLAD [21]; as well as the semantic-based algorithm Forest [7]. We used three datasets to evaluate the performance of these algorithms and quantified the performance of each VPR algorithm in various scenarios using evaluation metrics from the precision–recall curve, including AUC (area under the curve), Recall@100%recision, and Precision@100%Recall.

In the second experiment, we evaluated the computational cost of our algorithm’s descriptor-encoding and matching module on a computer equipped with an Intel Core i7-10700KF processor and an NVIDIA RTX 3050 GPU and compared it with several other algorithms. This computer was purchased at an ASUS store in Shenyang, China.

In the third experiment, we tested three different image processing approaches on the subsequence Slice8 of the Extended-CMU Season dataset. These four approaches include:

1. Using only Forest image descriptors for matching;
2. Combining SuperPoint-VLAD with Forest image descriptors to incorporate appearance information and construct Semlook local descriptors, followed by matching;
3. Using Semlook global descriptors for initial frame selection and then Forest image descriptors for matching;
4. Using Semlook global descriptors for initial frame selection and then using Semlook local descriptors for matching.

By conducting tests on the Extended-CMU Season dataset, we were able to evaluate the impact of different image processing approaches on image matching performance.

## 4. Results and Discussion

### 4.1. Analyzing VPR Performance

**Figure 6:**
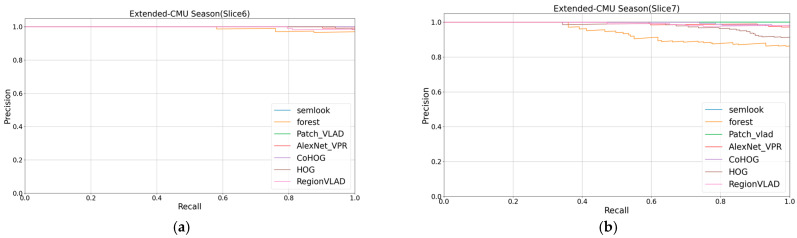
(**a**,**b**) show the precision–recall curves for all 7 VPR algorithms on the 2 datasets.

**Figure 7:**
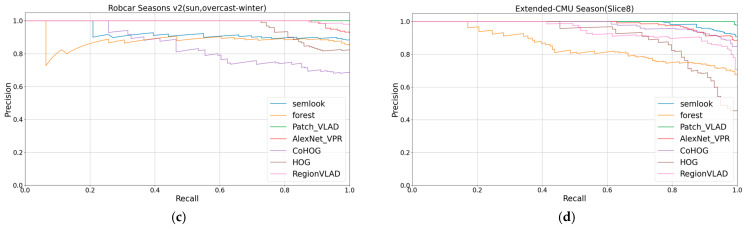
(**c**,**d**) show the precision–recall curves for all 7 VPR algorithms on the 2 datasets.

**Figure 8:**
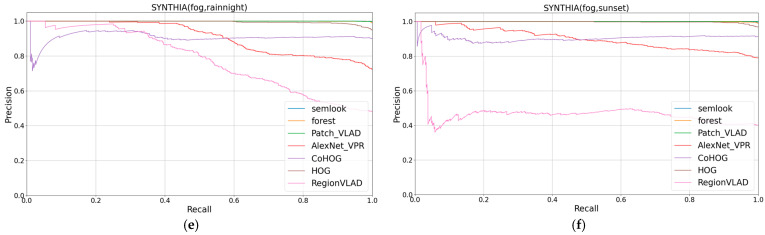
(**e**,**f**) show the precision–recall curves for all 7 VPR algorithms on the 2 datasets.

The study demonstrated the competitive performance of the Semlook-based visual place recognition (VPR) algorithm on the test dataset by evaluating and comparing VPR algorithms based on appearance, semantics, and deep learning on the same computer. The algorithm exhibited robustness to variations in lighting, seasons, viewpoints, and appearances. The evaluation metrics from Table 1 were used for quantification, and the visualization of these metrics is presented in Figure 6, Figure 7 and Figure 8,. The research findings indicate that the Semlook-based VPR algorithm achieves high accuracy in image matching tasks. It can handle diverse changes in different scenarios, demonstrating its strong performance in practical applications.

According to our experimental results, in terms of the AUC value, our method surpassed Forest, CoHOG, HOG, AlexNet_VPR, and Region VLAD on the Extended-CMU Season and SYNTHIA datasets. Our method demonstrated significant advantages in both regular and highly variable scenes. This achievement is attributed to the coarse-to-fine retrieval mechanism we employed. This mechanism not only includes preliminary selection based on semantic contours, but also involves fine selection that tightly integrates robust appearance information with semantic information. By narrowing the matching range through semantic contours and leveraging geometric relationships between different semantic categories as well as robust appearance information, we enhanced the expressive power of descriptors, making them more accurate and robust in the face of appearance and viewpoint changes. Additionally, our method achieved a performance comparable to Patch-NetVLAD. In datasets such as Extended-CMU Season_Slice6, Extended-CMU Season_Slice7, and SYNTHIA02_(fog, rainnight), both Patch-NetVLAD and our method reached a measurement value of 1.0, indicating that these two methods reached the upper limit of performance for the VPR method. In the SYNTHIA02_(fog, rainnight) dataset, our method slightly outperformed Patch-NetVLAD. However, in datasets like Extended-CMU Season_Slice8 and SYNTHIA02_(fog, sunset), our method slightly lagged behind Patch-NetVLAD. Meanwhile, Patch-NetVLAD has a higher computational cost. Considering that our method aims to design lightweight image descriptors for rapid operation on small robots, this is inconsistent with the high computational cost of Patch-NetVLAD. Finally, it is noteworthy that our method exhibited a slight decline in performance in the RobotCar Seasons V2 dataset. This may be due to the presence of a large amount of semantic segmentation noise in this dataset, which affected the performance of our method in this scenario. However, the experimental results show that, by further introducing appearance information based on Forest, which only uses semantic information, our method achieved a significant performance improvement.

Based on the experimental results, our method achieved advanced performance in two metrics: Precision@100% recall and Recall@100% precision, with average values reaching… Among them, Recall@100% precision plays a crucial role in SLAM systems. This is because, in the loop-closure detection process, the impact of incorrect loop closures far exceeds that of undetected loop closures, as incorrect loop closures can lead to bias in global optimization. Therefore, a high level of Recall@100% precision allows our method to be effectively applied in SLAM systems.

### 4.2. Computational Cost Analysis

Calculating cost is one of the critical indicators for evaluating the performance of VPR algorithms. According to the experimental results in Table 2, our algorithm demonstrates relatively high real-time performance in both the descriptor-encoding and matching stages. Specifically, the processing time for the entire Semlook descriptor was 54.55 ms, while the time cost using only the Semook global descriptor for preliminary selection was just 3.71 ms. Additionally, the time cost for using only the Semook local descriptor was 51.30 ms. From the perspective of time cost, the coarse-to-fine image matching strategy we employed has proven to be effective. When compared to other algorithms, we first contrasted it with appearance-based algorithms. We analyzed the experimental results and found that using the Semlook global descriptor for preliminary selection was faster than appearance-based algorithms such as CoHOG and HOG. Compared to semantic-based algorithms like Forest, we have introduced a primary selection strategy that is more convenient and efficient than matching all images. Our algorithm exhibits significant advantages regarding encoding cost and matching speed compared to deep learning-based algorithms such as RegionVLAD, AlexNet_VPR, and PatchNetVLAD. Our matching speed has been improved by several tens of times, allowing us to process more images within the same amount of time. Therefore, considering factors such as accuracy, robustness, and computational cost, our algorithm demonstrates a certain level of competitiveness compared to other algorithms.

### 4.3. Impact of Different Image Encoding Techniques on Performance

**Figure 9:**
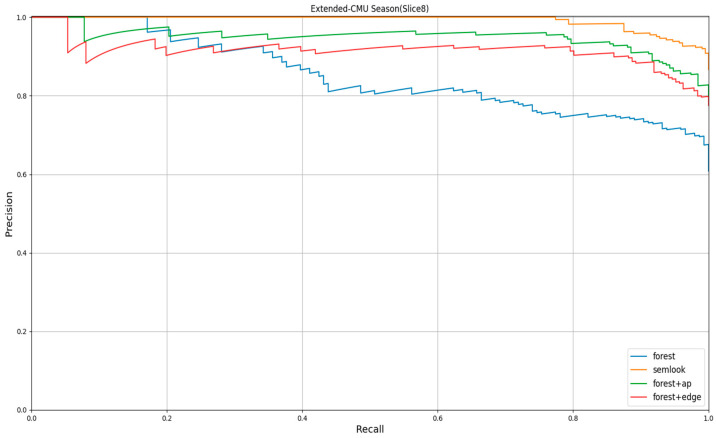
The impact of different treatments on performance.

Based on the experimental results for the image matching task on the Extended-CMU Season dataset, we compared the performance metrics of different image processing techniques, as shown in Figure 9, and drew the following conclusions:

Firstly, the Semlook global descriptor was used to perform initial filtering on candidate frames, followed by matching with the Semlook local descriptor. The results showed that this algorithm achieved the best performance in terms of the AUC value, Precision@100% recall rate, and Recall@100% accuracy. This indicates that the Semlook descriptor can improve image matching accuracy and recall rate when combined with appearance information and initial filtering strategies.

Next, by combining SuperPoint-VLAD to introduce the appearance information of images for constructing Semlook local descriptors and performing matching, a good performance was also achieved. Additionally, using Forest image descriptors for matching after the initial screening of candidate frames with Semlook global descriptors, although slightly inferior to the second approach in terms of performance, still outperformed the use of Forest image descriptors alone.

In conclusion, the experimental results demonstrate that both the strategy of using global descriptors for preliminary frame selection and incorporating appearance information into Forest image descriptors can improve the retrieval accuracy and recall rate.

## 5. Conclusions

The paper introduces an appearance-semantic descriptor, SemLook, consisting of global and local descriptors. The global descriptor is obtained by introducing a preliminary selection strategy based on semantic edges, which effectively excludes factors causing interference by eliminating objects with blurry outlines or indistinct shapes, thereby improving the algorithm’s accuracy and enhancing matching efficiency. The local descriptor is generated by fusing image appearance and semantic information, utilizing SuperPoint feature points combined with semantic images to classify and cluster the feature points, creating a VLAD vector representing each semantic contour, and assigning appearance information to each semantic object. This approach enhances the robustness and accuracy of the local descriptor while capturing the semantic and appearance features of objects in the image. Experimental results on public datasets demonstrate that the VPR algorithm based on SemLook is competitive in terms of robustness, accuracy, and computational efficiency.

## Figures and Tables

**Table 1:** The experimental results of the following seven comparison algorithms in three datasets.

Datasets	Ours	Forest	Patch-NetVLAD	AlexNet	RegionVLAD	HOG	CoHOG
Extended-CMU Season (Slice6)	1.0/1.0/1.0	0.99/0.97/0.58	1.0/1.0/1.0	0.99/0.99/0.90	0.99/0.98/0.80	0.99/0.98/0.94	1.0/1.0/1.0
Extended-CMU Season (Slice7)	1.0/1.0/1.0	0.93/0.86/0.36	1.0/1.0/1.0	0.99/0.97/0.59	0.99/0.98/0.47	0.98/0.91/0.34	0.99/0.98/0.74
Extended-CMU Season(Slice8)	0.99/0.91/0.78	0.85/0.68/0.17	0.99/0.97/0.99	0.98/0.88/0.62	0.95/0.70/0.41	0.91/0.45/0.45	0.97/0.84/0.61
RobotCar Seasons v2 (Sun, Winter)	0.93/0.88/0.21	0.88/0.85/0.06	1.0/1.0/1.0	0.99/0.93/0.88	0.99/0.97/0.87	0.96/0.82/0.72	0.84/0.68/0.25
SYNTHIA (Fog, Rainnight)	0.99/0.99/0.98	0.99/0.99/0.84	0.99/0.99/0.95	0.90/0.72/0.23	0.77/0.48/0.05	0.99/0.94/0.59	0.90/0.90/0.01
SYNTHIA (Fog, Sunset)	0.99/0.99/0.83	0.99/0.98/0.83	1.0/1.0/1.0	0.89/0.79/0.06	0.47/0.40/0.02	0.99/0.96/0.52	0.90/0.91/0.01

**Table 2:** The computational cost of each module (max/average/min).

Moule (ms/Frame)	Semlook	Edge (Coarse)	Foest + AP (Fine)	Forest	Region VLAD	AlexNet_	Patch-NetVLAD	HOG	CoHOG
	Three Datasets	Three Datasets	Three Datasets	Three Datasets	Three Datasets	Three Datasets	Three Datasets	Three Datasets	Three Datasets
Encoding	68.41/54.46/40.99	9.50/3.64/3.07	58.91/50.82/37.92	40.3/32.6/25.9	1384.6/1135.9/918.4	1625.7/681.4/517.8	526.1/415.3/375.7	11.5/4.84/3.67	270.9/113.7/86.3
Matching	0.16/0.09/0.04	0.12/0.07/0.03	0.67/0.48/0.31	0.58/0.34/0.21	0.19/0.085/0.07	771.1/379.6/211.8	108.3/49.7/41.6	0.14/0.07/0.04	3.23/1.72/0.96
Total	68.57/54.55/41.03	9.62/3.71/3.10	59.58/51.30/38.23	40.8/32.9/26.1	1384.7/1136.9/918.5	2396.8/106.1/729.6	964.8/465.8/417.5	11.6/4.91/3.71	274.1/115.5/87.3

## Data Availability

Data are contained within the article.
